# The effect of pollutant exposure on cough in progressive pulmonary fibrosis

**DOI:** 10.5588/ijtldopen.25.0427

**Published:** 2025-10-10

**Authors:** H. Vander Linden, U. Zanini, M. Kalluri, J. Cole, P. Boulanger, M. Feist, G. Ferrara

**Affiliations:** ^1^ Naiad Lab, Edmonton, AB, Canada;; ^2^ Department of Medicine and Surgery, University of Milan-Bicocca, SC Pneumologia, Fondazione IRCCS “San Gerardo dei Tintori”, Monza, Italy;; ^3^ Division of Pulmonary Medicine, Department of Medicine, Faculty of Medicine and Dentistry, Edmonton, AB, Canada;; ^4^ Digital Health Unit, Clinical Trial Office, University of Alberta, AB, Canada;; ^5^ Faculty of Computing Science, University of Alberta, Edmonton, AB, Canada.

**Keywords:** PM_2_._5_, ozone, interstitial lung disease, cough episodes

Dear Editor,

Cough is a prevalent respiratory symptom affecting nearly one tenth of Americans, and its severity is exacerbated by air pollution.^1^ Among patients with interstitial lung disease (ILD), cough is a common symptom that can significantly impact quality of life and is associated with both mortality and disease progression.^2^ Cough assessment relies on questionnaires and clinical evaluation, but these methods have limitations.^3^ Air pollution has emerged as a leading risk factor for death globally.^4^ Our study aimed to analyze the effect of the most common pollutants on cough in progressive pulmonary fibrosis (PPF) using a wearable for objective cough measure.^5^

This was a single-centre, prospective cohort study enrolling PPF patients. Cough was recorded for 6 months with ADAMM-RSM™, a wearable device made of soft plastic. The participants applied the device to their chest in the morning using adhesive tape and charged it at night. Participants were assessed in person at baseline, 3, and 6 months. Monthly phone calls addressed any technical issues with ADAMM-RSM™ while monitoring for significant adverse events. We explored how often an air pollutant spike occurred before each spike in cough. Cough data collected from the device was cleaned so the analysis only considered data recorded during the hours the device was being worn. Data on hourly levels of PM2.5, ozone, and wind speed, coupled with the participant’s postal code, were obtained from public databases (https://www.alberta.ca/access-air-data). The use of ADAMM-RSM™ in this study received an investigational testing authorization (ITA, protocol N. ePReBMs-01-2019, App. N. 318122) by HealthCanada. The clinical trial was approved by the University of Alberta Review Ethics Board (PRO00097162) and registered on clinicaltrials.gov (NCT04857814). All patients signed informed consent before enrolling in the clinic trial.

A cross-correlation using the cough count and air quality data showed a weak correlation after a few days (data not shown). Due to the difference in the degree to which participants reacted to the pollutants in the air, simplifying the data allowed for better comparison across all participants. Instead of comparing the raw cough counts and air quality levels, the data were grouped into high cough counts and high levels of pollutants, referred to as spikes. In other words, significant upward changes in the data were marked, and mathematically, the spikes were calculated as follows. A spike in cough/hour was a data point in the top 10% of a patient’s coughing data, representing the highest value within a 24-hour window for each study participant. To increase the stringency of the analysis, cough spikes that did not meet a certain threshold were excluded. The spikes had to be either more than 25% of the maximum cough spike of the patient and exceed the patient's average cough/ hour by over 3 standard deviations, or it had to be more than 50% of the patient’s maximum spike recorded during the study. This ensured each spike used in the analysis was a significant increase from each patient's average cough/hour. For air pollution, a spike was a point in the top 1.5% of data collected during the study period by a patient's closest weather station. The maximum spike within 7 days was selected. To increase the stringency of the analysis, air pollutant spikes had to be either more than 25% of the maximum spike ever recorded during the study and 4 standard deviations above the study average values, or greater than 50% of the maximum patient’s weather station spike. Missing data were excluded.

A total of 8 patients entered the study and 2 dropped out (1 due to a skin reaction to the tape used to secure the device, while the other never wore the device). Data from 6 participants, collected over 1,170 days of monitoring, recorded 24,371 coughs and 24 cough spikes. Total cough counts per patient ranged from 2,093 to 6,697, with device usage varying from 43.4%–85.6%. The total number of cough spikes was 24 in the whole cohort, with a range from 2–6 per patient. Most of the spikes in PM 2.5 and ozone preceded an increase in cough, with 27 out of 29 (93%) of the PM. 2.5 spikes and 26 out of 29 (90%) of ozone spikes preceding a cough spike, respectively. Average PM2.5 values and standard error of the mean (SEM) at baseline and during peak recordings were 6.9±0.1 mcg/m^3^ and 58.9±6.8 mcg/m^3^, respectively. Similarly, average ozone levels at baseline and during peak recordings were 22.8±0.1 ppb and 51.4±1.2 ppg, respectively. Wind speed varied from 7.5±0.03 Km/h at baseline to 27.3±0.59 Km/h at peak recordings. Average cough/hour during the study increased from an average of 9.6±0.6 coughs per hour to 26.4±3.7 at peak recordings.

The data showed a higher frequency of PM2.5 spike in the 7–14 days preceding a significant increase in coughing (Figure panel A). The strongest relationship was observed for day 8: during this time, PM2.5 spikes were 2.05 times more frequent (95% CI: 1.39–2.69) before a cough spike compared to baseline. The most statistically significant relationship was observed over 11 days, with a p-value=0.0003 and a power 0.93. Also, ozone spikes were associated with a pronounced increase in cough spikes (Figure panel B). The strongest relationship between cough and ozone spikes occurred within the first 5 days. Ozone spikes were 2.1 times more frequent before a cough spike, indicating a significant acute response (95% CI: 1.279–2.943). This strong effect gradually diminished, returning closer to baseline levels by day 10. As a control, wind speed spikes did not correlate with an increase in cough spikes (Figure panel C).

**Figure.**
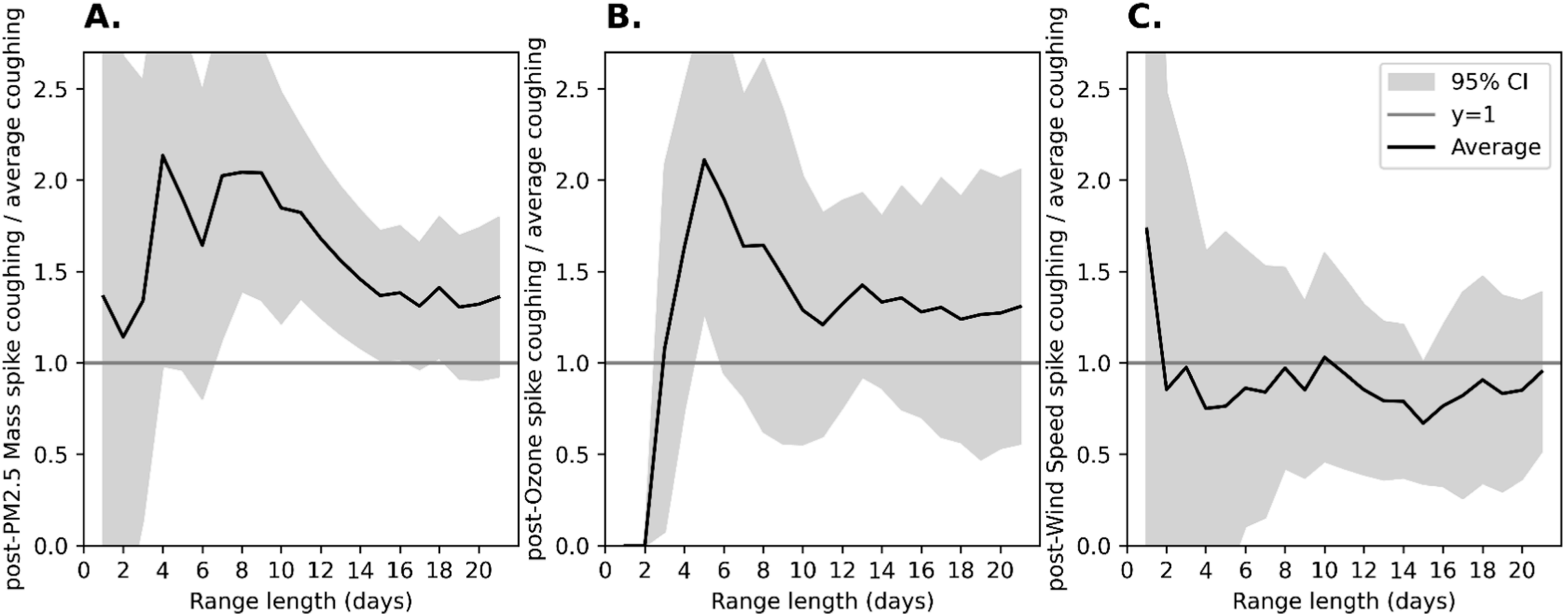
Ratio of post exposure-peak coughing to average coughing as a function of range length (days), for various environmental factors. **A:** PM_2_._5_; **B:** ozone; **C:** wind speed.

This is the first study to examine cough in patients with PPF by analyzing it over a 6-month period with a wearable, and to explore its correlations with PM2.5 and ozone data. PM2.5 levels significantly impacted cough episodes 7–14 days following exposure, suggesting subacute inflammatory or irritative mechanisms. Ozone spikes were associated with an acute increase in coughing episodes within 4 days, followed by a return to baseline by day 10. These results are in keeping with clinical observations from retrospective cohort studies, indicating increased risk of exacerbation and admission exactly in the same timeframes.^6^ These results underline the importance of considering the effect of environmental exposures in epidemiological and clinical studies on cough. Our results underscore the importance of integrating environmental data as an independent variable in clinical trials focusing on new interventions for cough in PPF and other respiratory disease.

The study has several significant limitations: the small sample size, variability in adherence to the wearable device, and the potential influence of unmeasured confounders such as concomitant therapies or comorbidities. Despite the small number of participants, prospective collection and stringent data analysis, with time-to-event association similar to the timelines of clinical outcomes noted in retrospective epidemiological studies provide robustness to our results.

In conclusion, our findings support the hypothesis that PM_2_._5_ and ozone levels are associated with an increase in cough episodes among patients with PPF, suggesting potential mechanisms of acute and subacute inflammatory response. Moreover, large cohort studies have shown that environmental exposures may contribute to acute exacerbations, and cough monitoring could thus serve as a novel indicator of climate-related stress on the respiratory system.^7^ Future research with larger, multicenter cohorts will be needed to confirm these results.
